# The association between eating disorders and mental health among workers

**DOI:** 10.1192/j.eurpsy.2023.909

**Published:** 2023-07-19

**Authors:** I. Sellami, A. Abbes, A. Hrairi, M. A. Ghrab, A. Meska, M. Tah, M. L. Masmoudi, K. Jmal Hammami, M. Hajjaji

**Affiliations:** 1 occupational medecine, Hedi Chaker hospital, university of Sfax; 2 occupational medecine, University of Sfax; 3 occupational medecine, Hedi Chaker Hospital, Sfax, Tunisia

## Abstract

**Introduction:**

Eating disorders are serious medical conditions labelled by severe disturbances to one’s eating behaviours. These disorders can affect employees’ physical and mental health.

**Objectives:**

This study aimed to evaluate the association between eating disorders and workers’ mental health.

**Methods:**

The study was conducted among a group of workers from an electricity society. Data were gathered between January-June 2022 using a self-administered questionnaire including socio-professional characteristics, eating disorders evaluation, and mental health assessment using the Hospital anxiety and depression scale (HADS).

**Results:**

Our study included 92 workers. Their mean age was 43.8 ± 11.7 years and 73 were male (79.3%). The average job tenure was 17.6 ± 11.2 years. Thirty-eight participants (41.3%) had eating disorders. With the reference to HADS, 47 (51.1%) and 16 (17.4%) participants presented symptoms of anxiety and depression, respectively. Regarding the patients’ anxiety levels, it was found that 82.6%, 12%, and 5.4% appeared to have mild, moderate, and severe anxiety, respectively. As for the depression levels of patients, 45 were mildly depressed (48.9%), 31 were moderately depressed (33.7%), and 16 were severely depressed (17.4%). Participants with eating disorders had higher scores of anxiety and depression p=0.001 and p=0.003 respectively.

**Conclusions:**

According to our study, eating disorders had a significant association with the mental health of workers. Therefore, it is important to provide training for workers to have a healthy balanced diet to guarantee good mental health.

**Disclosure of Interest:**

None Declared

